# Automatic measurement of epithelium differentiation and classification of cervical intraneoplasia by computerized image analysis

**DOI:** 10.1186/1746-1596-5-7

**Published:** 2010-01-22

**Authors:** Michel Jondet, Régis Agoli-Agbo, Louis Dehennin

**Affiliations:** 1 Cabinet de Pathologie, 34 Rue Ducouedic, 75014 Paris, France

## Abstract

**Background:**

The feasibility of evaluating an objective grading of cervical intraneoplasia lesions (CIN) is attempted using an automatic computerized system able to measure several valuable parameters with special reference to epithelium differentiation.

**Methods:**

4 groups of 10 images each were selected at random from 68 consensus images coming from 80 archival cervical biopsies, normal (n = 10), CIN 1 (n = 10), CIN 2 (n = 10), CIN 3 (n = 10). Representative images of lesions were captured from the microscopic slides and were analyzed using mathematical morphology, with special reference toVoronoï tessellation and Delaunay triangulation. Epithelium surface, nuclear and cytoplasm area, triangle edge and area, total and upper nuclear index were precisely measured in each lesion, and discriminant coefficients were calculated therewith. A dilation/erosion coefficient was automatically defined using triangle edge length and nuclear radius in order to measure the epithelium ratio of differentiation. A histogram ratio was also automatically established between total nuclei and upper nuclei on top of differentiated epithelium. With the latter two ratios added to the nucleo-cytoplasmic ratio, a cervical score able to classify CIN is proposed.

**Results:**

There is a quasi-linear increase of mean cervical score values between normal epithelium and CIN 3: (27) for normal epithelium, (51) for CIN 1, (78) for CIN 2 and (100) for CIN 3, with significant differences (P < 0.05).

**Conclusion:**

Our results highlight the possibility of applying a cervical score for the automatic grading of CIN lesions and thereby assisting the pathologist for improvement of grading. The automatic measure of epithelium differentiation ratio appears to be a new interesting parameter in computerized image analysis of cervical lesions.

## Introduction

Cervical intraepithelial neoplasia (CIN) has aroused a lot of interest with regard to classification. The world-wide classification, proposed by Richart [1], is based on several morphologic criteria in order to predict the biological behavior of abnormal epithelium. Among them, the most reliable features are: 1 - degree of epithelium differentiation, 2 - nucleus to cytoplasm ratio, 3 - cellular maturation, 4 - nuclear analysis (maturation and mitotic activity). When scrutinizing the literature, it appears that a strong consensus exists on the diagnosis of normal epithelium and high grade lesions, but problems appears mainly with the classification of CIN 1 and CIN 2 due to poor inter- and intra-observer correlations [2-8]. A new classification introducing low grade (HPV infections + CIN 1) and high grade (CIN 2 to carcinoma in situ) represents a cut-off, pointing out patient treatment in the high grade group only.

Several attempts have already been made to automatically and objectively grade CIN using image analysis. Based on mathematical morphology Keenan et al. [9] focused on morphology and Guillaud et al. [10] paid more attention on nuclear features. An automatic stereologic measurement using Ki67 marker has been proposed by Kruse et al [11]. Nevertheless, these techniques still remain of poor applicability due to difficulties with their implementation. Following up these original reports, we have developed a similar procedure taking into account a limited number of parameters and we propose a new concept able to automatically measure the degree of epithelium differentiation.

## Methods

### Tissue processing

Archival cervical biopsies came from 80 patients (age 25 - 43 years) undergoing punch biopsy during colposcopy, after detection of cytological abnormalities in cervical smear. Cervical tissues, which were treated with formaldehyde-acetic acid fixative and embedded in paraffin, were cut into 2.5-μm serial sections. Sections were deparaffined and stained automatically (Autostainer, Microm, Germany) with a modified Weigert stain [MJ], listed on table 1, thereby enhancing nuclear membrane and general contrast.

**Table 1:** Weigert-Lead stain (M. Jondet)

Staining solutions	
1 - Weigert's iron haematoxylin	
a - Haematoxylin solution	
Haematoxylin	1 g
Ethanol	100 ml
	
b - Iron solution	
30% Ferric chloride solution in water	4 ml
Hydrochloric acid 1 M	1 ml
Distilled water	95 ml
Mix equal volumes of solution a and b, use within 60 min	
	
2 - Lead citrate	
lead nitrate	1.33 g
sodium citrate	2.10 g
distilled water	30 ml
Add sodium hydroxyde 0,4 M untill precipitate dissolves	
	
Method	
a - dewax and rehydrate sections	
b - Weighert's haematoxylin	1 minute
c - wash with distilled water	
d - saturated picric acid in water	1 minute
e - wash with distilled water	
f - lead citrate	1 minute
g - wash with water	
h - mount in Eukitt after alcohol and xylene clarification	
	
Results	
Nucleus	Black
Nuclear membrane	dark black
Cytoplasm	light grey

### Image capture

Microscopic slides were observed with a Zeiss Axioskop microscope (Zeiss, Göttingen, Germany) with a ×25 magnification (Zeiss Plan-Neofluar 25/0.8 lens) under standard illumination (same voltage and diaphragm aperture). For each biopsy, at least 1 image was recorded. In some samples, where different grades of lesion were observed on the same biopsy, a microscopic field of each grade was recorded. The images were recorded with a black and white CCD camera (model PRO-110SP, Apro Media, South Korea), they were digitized (472 × 608 pixels, 256 grey level) by an electronic video card (ATI-All in Wonder 128, ATI Technologies Inc., Ontario, Canada) and recorded in bitmap format. Each image had a 286,976 pixel area, corresponding to a 0.066 mm^2 ^real surface on the microscopic slide, so that 1 pixel corresponded to 0.22 × 10^-6 ^μm^2^.

A total of 86 microscopic fields, corresponding to normal and cervical lesions of different grades, were submitted to 4 gynaeco-pathologists for classification, one of them being the first author [MJ]. Images were submitted to the 4 pathologists independently, and the same images were viewed again by each one 3 months later, independently of the first vision. The pathologists were asked to grade the images in 4 groups (normal, CIN 1, CIN 2 and CIN 3). An intra- and inter-observer consensus was established with 68 images, among which 10 images were selected at random in each group.

### Image analysis

#### a - software

Micromorph 1.4 version image analysis system (Mathematical Morphology Software CMM-ENSMP, Paris, France) was used for image processing. This software was developed for the use of mathematical morphology and contains nearly all the tools of this image analysis procedure [12]. Some features which are lacking in the original software, such as Delaunay triangulation, have been developed by RA [13]. Automatic algorithms for the measurement of epithelium differentiation area and nuclei histogram have been developed by MJ and RA, with specific automation in order to perform calculations on large series of images.

#### b - Manual and semi-automatic procedures

Each gray image was manually processed with Photoshop 5.5 in order to select the original epithelial tissue, thereby eliminating artifacts and stroma. The basal lamina and the surface of the epithelium were delineated, before running image analysis in the region of interest (ROI), delimited in that way. The cleared image was then treated by the image analysis system for segmentation of nuclei. After increasing light and contrast, the image was submitted to thresholding, followed by proper segmentation. A 1 pixel border was added to the final image and the non-tissue areas were filled by superposition of the original image with the segmented one. With such a procedure, the nuclei cutting the image edge were suppressed, allowing a precise measurement of ROI and average nuclear areas.

#### c - Automatic procedure

**Figure 1:**
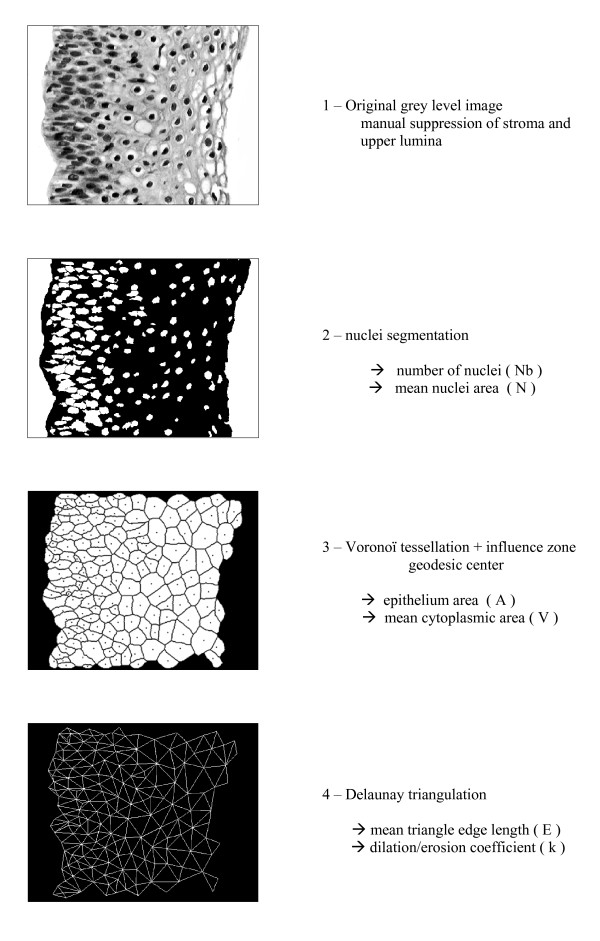
**The image analysis process till Delaunay triangulation**.

Once the segmentation was properly achieved, an automatic algorithm provided several measurement parameters. A Voronoi tessellation was done delimiting the zone of influence on nuclei. This procedure gave an approximation of the cytoplasmic area, considering that squamous epithelium is made of joint cells. The geodesic center of each zone of influence was then used to proceed towards a Delaunay triangulation, with subsequent retrieval of the mean edge length of the triangles. The procedure is represented on figure 1.

**Figure 2:**
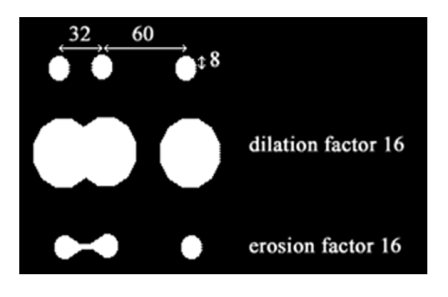
**Calculation of the dilation/erosion coefficient**.

The epithelial differentiation, representative of the CIN definition, has been introduced by Richart [1] who arbitrarily separated the cervical epithelium in 3 tier, with a subsequent classification corresponding to the evolution of the lesion. This grading was based on the proportion of basaloid undifferentiated cells which nuclei were getting close together as the lesion increased. Mathematical morphology is well adapted for the definition of this particular item. Using a combination of dilation and erosion procedures, it was possible to automatically measure this degree of differentiation, as illustrated by the figure 2: the 3 particles have a 199 pixel area (= 8 pixel radius), the middle particle being separated from its neighbors by 32 and 60 pixel. Dividing this distance by 1/4th of the radius gives respectively values of 16 and 30 pixel. Applying a dilation value of 16 on the image allows the 2 nearest neighbors to merge. A subsequent erosion with the same value restores the original image but the nearest neighbors are still merged. In accordance with the preceding theory, this simple calculation can be applied to cervical epithelium as the distance between the nuclei's nearest neighbors is shorter in differentiated epithelium when regarding the vertical evolution of the lesion than in normal epithelium.

**Figure 3:**
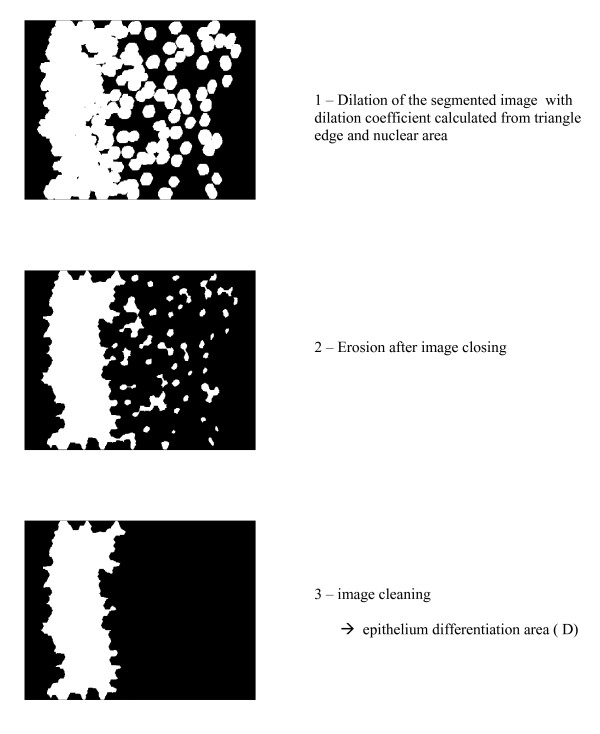
**Measurement of the differentiated epithelium**.

The dilation/erosion coefficient was established from the mean value of the triangle edge and the mean nuclear area measured in the 4 groups and defined by dividing the mean triangle edge by 1/4th of the mean radius of the nucleus, which was achieved automatically by the vision machine. Some other mathematical tools, such as hole closing and cleaning, were applied in order to afford a properly designed image for measurements. The final result is represented on figure 3.

**Figure 4:**
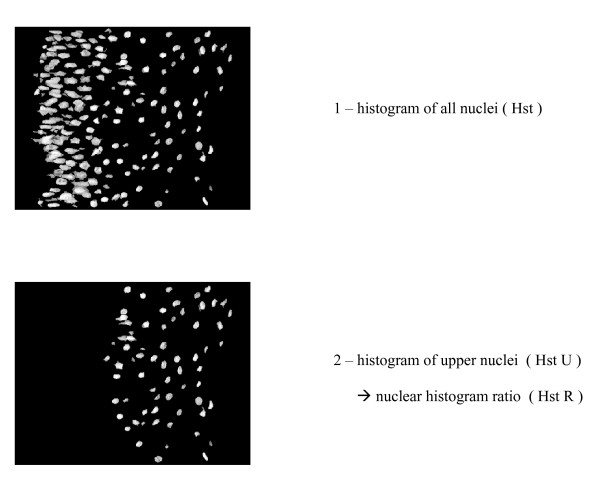
**Measurement of the nuclei histogram (total and upper nuclei)**.

By superimposition of the binary segmented nuclei on the original grey level image and inversion of the resulting image, it was possible to measure the histogram corresponding to all nuclei. The same procedure was applied to the non-differentiated epithelium giving the nuclei histogram value in the upper part of the epithelium. This is represented on figure 4.

The following parameters were automatically measured by the vision machine on the resulting images:

1 - area of the selected epithelium (A)

2 - mean area of the nuclei (N)

3 - mean area of the zone of influence (V)

4 - epithelial differentiation (D)

5 - total nuclei histogram (Hst)

6 - upper nuclei histogram (HstU).

From these 6 parameters, 3 ratios were inferred:

- nucleo-cytoplasmic ratio (N/V) = ratio of nucleus area to cytoplasmic area;

- epithelial differentiation ratio (DR) = ratio of differentiated epithelium area (D) to total epithelium area (A);

- upper nuclei histogram ratio (HstR) = ratio of upper nuclei histogram (HstU) to total nuclei histogram (Hst).

The 3 discriminant ratios were used to derive a mathematical score, in order to get a near to 100 value for high grade lesions (CIN 3).

The same calculation was applied for all groups of images.

### Statistics

Distributions have been found normal, except for 2 out of 40 data (10 values in each of the 4 groups under study). Therefore comparisons between 2 successive groups (normal *vs *CIN1, CIN 1 *vs *CIN 2, CIN 2 *vs *CIN 3) have been made by t-test, with Welch correction when unequal variance was assumed. The levels of significance are P < 0.05. GraphPad Prism 5.02 (GraphPad Software Inc., La Jolla, California, USA) was the statistical software used.

## Results

On table 2 are presented the data obtained in the 4 groups for 6 basic parameters:

**Table 2:** Means (± SD) of significant features measured in the 4 diagnostic groups (values are expressed in pixel)

	Normal	CIN 1	CIN 2	CIN 3
Epithelium area (A)				
mean	212018.0	211203.0	193261.0	183499.0
sd	25165.0	19507.0	34755.0	36993.0
Nucleus area (N)				
mean	173.9	239.1	200.6	206.7
sd	33.8	50.7	41.5	37.0
Cytoplasm area (V)				
mean	1740.3	995.5	595.1	442.3
sd	277.5	170.0	114.8	37.4
Epithelium differentiation (D)				
mean	30136.3	75070.4	111642.0	157383.9
sd	4066.0	10032.7	27981.4	32505.0
Total nucleus histogram (Hst)				
mean	19.1	46.7	59.5	75.6
sd	5.3	8.2	9.7	15.2
Upper nucleus histogram (HstU)				
mean	7.3	13.4	15.6	3.5
sd	2.2	3.0	3.8	2.5

- mean epithelium area (A) decreases without statistical significance;

- mean nucleus area (N) increases significantly from normal to CIN 1 and remains further at the same level for high grade lesions;

- mean cytoplasm area (V) decreases from normal up to CIN 3;

- mean epithelium differentiation (D) increases significantly from normal up to CIN 3;

- total nucleus histogram (Hst) increases significantly from normal up to CIN 3;

- upper nucleus histogram (Hst U) increases significantly from normal to CIN 1, then without significant difference between CIN1 and CIN 2, and with a significant decrease between CIN 2 and CIN 3 due to a drastic diminution of non differentiated epithelium in CIN 3 (by definition, nuclei maturation should be 100% within CIN 3).

Most of these results confirm the general impression when observing a cervical biopsy under the microscope. Of interest are the ratios calculated from data on table 2, which represent an additional 4 parameters:

- nucleo-cytoplasmic ratio (N/V) increases significantly from normal up to CIN 3;

- epithelium differentiation ratio (DR) increases significantly from normal up to CIN 3, and this means that the mathematical procedure is in accordance with pathology. Measurements have been made on the nuclei and did not take into account the cytoplasm, thereby resulting into ratios which do not reach 100%, as expected. This is due to the fact that epithelium differentiation was measured with nuclei area and did not take into account cytoplasm area, although present, but in a small amount on the upper side of the basal part of epithelium;

- upper nuclei histogram ratio (Hst R) decreases with increasing epithelium differentiation from normal up to CIN 1, with a drastic fall for CIN 3 due to the low number of nuclei in the upper part.

Finally, the cervical score, which represents a calculation from the preceding ratios, displays a quasi-linear and significant progression from normal up to CIN 3 lesions with no overlapping between the 4 groups.

**Figure 5:**
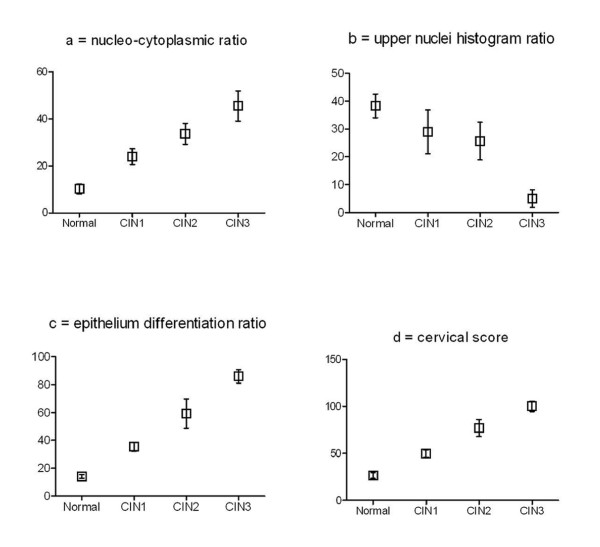
**Data on ratios (mean ± SD).** Statistical comparisons for each ratio (Normal *vs *CIN1; CIN1 *vs *CIN2; CIN2 *vs *CIN3) are significant (P < 0.05), except for upper nuclei histogram ratio (CIN1 *vs *CIN2).

The 3 preceding ratios and the cervical score are represented on figure 5.

## Discussion

A vision machine is able to measure a nearly unlimited number of parameters when analyzing an image, but we completely agree with Keenan [9] who pointed out that the development of a mechanical vision system is complicated.

The purpose of this work was to limit the number of parameters by retaining only those values which are as close as possible to the parameters taken into account when a pathologist analyses and grades a cervical biopsy. With a specific algorithm we were able to record automatically 6 reliable parameters and to establish a cervical score for grading objectively a cervical biopsy. We took into account the original classification proposed by Richart [1] which is still the gold standard in the field and pointed out the epithelial differentiation parameter.

Image captures were done with a ×25 objective which suits perfectly to our optical system, allowing maximum vision of total epithelium height, especially in the normal group. The application of contrast staining appeared to be a valuable procedure in the segmentation process. Segmentation is a major problem in image analysis, as the nuclei are often superimposed, particularly in high grade lesions and cancer. Up to now there is no valid solution, because the manually segmenting of clusters is time consuming and subject to errors, particularly in high grade lesions and cancer. After several attempts to achieve a fully automatic process, it appeared that a manual or semi-automatic procedure was necessary to reach better segmentation before the automatic measurement of the pertinent parameters. This is a general problem in image analysis and represents an important issue for the standardization of image quality, as pointed out by Kayser [14,15].

Voronoï tessellation is based on nuclei segmentation and determines on each nucleus the zone of influence, which is a mathematical morphology item that suits well with biological observation, because the nucleus exerts an "influence" on the surrounding cytoplasm. Therefore, we can relate the zone of influence area to cytoplasm area. As proposed by Serra (Personal Communication), the Delaunay triangulation was then drawn from the geodesic center of each zone of influence, this procedure allowing a more accurate triangulation because it takes into account the nearest neighbors and eliminates false relation of 2 cells being far away from each other, when there is convexity in the epithelium for example.

The Delaunay triangulation measures the mean triangle edge length which was used to define the dilation/erosion coefficient. By combining this value with the mean value of nuclei area it became possible to automatically measure differentiated epithelium. An alternative for the measurement the dilation/erosion coefficient consist in the application of the minimum spanning tree technique giving rise to the minimum distance between the nuclei's nearest neighbors [16]. A specific algorithm has to be developed in our vision machine in order to check which is the adequate technique. Our procedure, using automatic dilatation/erosion makes it easier in cases of convexity or irregularities of the basal lamina or papillomatosis, which represents most of the cases encountered in routine pathology. Considering percentage differentiation, correlation with Richart's classification was found, though normal epithelium is made up of limited number of basal cells, not considered as differentiated upon microscopic observation, but displaying a small degree of differentiation with the vision machine.

Cell maturation in the upper part of the differentiated epithelium represents an important factor in the grading, especially in cases of koïlocytosis. Our staining procedure is based on iron-haematoxylin treatment. By increasing the nucleus contrast with our modified technique, although haematoxylin is not stochiometric *per se*, the intensity of the nucleic staining, which is taken into account in routine microscopy, can bring up an interesting data. When establishing the histogram of all nuclei on one end and the upper nuclei on the other end, the histogram ratio gives an adequate appreciation of nucleic chromaticism, independent of specimen thickness and staining intensity.

Nucleo-cytoplasmic ratio is an important data from the spatial point of view. In normal epithelium near the surface, the cytoplasm is very large and the nucleus becomes smaller during normal maturation. As the histological section is 2.5 μm thick, only a few nuclei are present with their surrounding cytoplasm, leading to a false estimation of nucleo-cytoplasmic ratios. Data are nevertheless comparable because they are objectively done by the machine and are reproducible.

We did not consider mitoses which is an important parameter in the classification. This can be achieved when images are captured with a ×1000 magnification [10]. Other parameters such as DNA ploïdy can be measured by image analysis [17,18], but they represent a more complicated approach and are time consuming. We also did not take into account the immuno-histochemistry with Ki65 and P16, which can represent an additional parameter [19]. Comparing data from the 4 pathologists, a good correlation was observed with the machine in 79% of the cases, with consensus cases taken into consideration for the selection of the 4 groups submitted to the vision machine. The data produced by automatic calculation display some statistical significance among the 4 groups, but the low number of cases in each group may be a limitation. Twenty six more images were added to the 86 previously analyzed, and were submitted to the 4 pathologists in a confrontation meeting without regard to the previous results. There was 77% correlation between the consensus diagnosis and the vision machine results which confirms the results of Keenan [9].

The use of mathematical morphology makes our algorithm transposable to any system based on that image analysis system, after proper calibration of the optical system specific to each user. The staining procedure, the microscopic field illumination and the automatic measures with our system were standard and gave reproducible results, these parameters being considered as very important [20].

The cervical score we proposed presents a quasi-linear progression from normal to high grade lesions and fits closely to Richart's classification. The problem however is to determine an adequate cut-off value for the decision to treat or not to treat a patient.

Though it may not always be comfortable to accept that a machine can be able to grade a CIN, we hope that our contribution to the proposal for a reliable scoring will help pathologists to deliver objective diagnoses, further more in experimental studies where objectivity and quantification are requested. So far, we consider that the vision machine is not able to produce a diagnosis *per se*, the selection of the optical field to be analyzed remaining operator dependent. Nevertheless it can help the pathologist to accurately establish the most valuable diagnosis. Although some artefacts may occur, precision seems fairly good, which is hardly the case when grading is done by routine observation on microscopic slides, as inter and intra variability is a limiting factor. The present investigation should be considered as a contribution to total morphologic analysis of any cervical lesion by measuring objective parameters, with special reference to epithelial differentiation.

## Conclusion

Using Voronoï tessellation and Delaunay triangulation on an image analysis system we were able to demonstrate that it is possible to grade automatically and precisely a cervical intraneoplasia lesion. This was achieved using a modified Weigert dye enhancing nuclear contrast and we were able to propose a cervical scoring. Our contribution to this difficult approach, compared to previous published results, was to add the automatic measurement of the pathologic epithelium differentiation. Our algorithms developed for this specific application can be adapted to any vision machine using mathematical morphology. More information can be made available by immuno-histochemistry combined with image analysis. We are looking forward pursuing further developments to this preliminary work in order to help pathologists to become more and more accurate and objective in their grading of cervical intraneoplasia.

## Competing interests

The authors declare that they have no competing interests.

## Authors' contributions

MJ initialized the study and drafted the paper. RA was involved in developping the specific algorithms and LD was involved in the statistical aspect and reviewing the manuscript. All authors read and approved the final manuscript.
